# Intestinal Microbiota in Early Life: Latest Findings Regarding the Role of Probiotics as a Treatment Approach for Dysbiosis

**DOI:** 10.3390/nu17132071

**Published:** 2025-06-21

**Authors:** Gabriel Florin Răzvan Mogoş, Monica Manciulea (Profir), Robert-Mihai Enache, Luciana Alexandra Pavelescu, Oana Alexandra Popescu (Roşu), Sanda Maria Cretoiu, Ileana Marinescu

**Affiliations:** 1 Department of Surgery, University of Medicine and Pharmacy of Craiova, 200349 Craiova, Romania; gabrielmogos@yahoo.com; 2 Department of Morphological Sciences, Cell and Molecular Biology and Histology, Carol Davila University of Medicine and Pharmacy, 050474 Bucharest, Romania; monica.profir@rez.umfcd.ro (M.M.); luciana.pavelescu@umfcd.ro (L.A.P.); oana-alexandra.rosu@rez.umfcd.ro (O.A.P.); 3 Department of Oncology, Elias University Emergency Hospital, 011461 Bucharest, Romania; 4 Department of Radiology and Medical Imaging, Fundeni Clinical Institute, 022328 Bucharest, Romania; robert-mihai.enache@rez.umfcd.ro; 5 Department of Psychiatry, Faculty of Medicine, University of Medicine and Pharmacy of Craiova, 200349 Craiova, Romania; ileana.marinescu@umfcv.ro; 6 Psychiatry Clinic, Craiova Clinical Hospital for Neuropsychiatry, 200615 Craiova, Romania

**Keywords:** gut microbiota, perinatal life, child microbiota, probiotics, dysbiosis

## Abstract

The gut microbiota plays a crucial role in early-life development, influencing various aspects of health and disease. Dysbiosis, an imbalance in the gut microbiota, has been linked to multiple health conditions, including allergies, asthma, and obesity. In early life, the gut microbiota plays a key role in the development and maturation of the immune system. Probiotics, live microorganisms that confer health benefits when administered in adequate amounts, have emerged as a potential treatment approach for dysbiosis in early life. Dysbiosis can alter the resistance to pathogens, promoting atopic diseases, food sensitization, and infections such as necrotizing enterocolitis (NEC). Probiotics have been shown to modulate the composition and function of the gut microbiota in the perinatal and infant periods. They can increase the abundance of beneficial bacteria, such as *Bifidobacteria* and *Lactobacilli*, and reduce the levels of potentially harmful bacteria. Not all probiotics are created equal. The effects of probiotics can vary depending on the specific strain used. Probiotics have also been investigated for their potential benefits in other areas of infant health, such as reducing the risk of respiratory infections and improving growth and development. This review aims to analyze the current data in the literature and to evaluate the health benefits of probiotic administration in early life. Several studies have investigated the use of probiotics in preventing or treating allergic diseases, such as eczema and food allergies. While some studies have shown promising results, more research is needed to fully understand the benefits and risks of probiotics in early life. In conclusion, using probiotics to prevent dysbiosis-related conditions may be considered a method of ‘programming’ the individual for optimal health maintenance.

## 1. Introduction

The human gut microbiota plays a pivotal role in maintaining health and contributing to disease, beginning as early as the perinatal period. A complex interplay of factors, including maternal diet, gestational age, the mode of delivery, feeding practices, and exposure to antibiotics, influences its composition and development [1,2,3,4]. Disruptions to this balance, known as dysbiosis, have been implicated in a wide range of pediatric conditions, such as allergies, autoimmune disorders (e.g., type 1 diabetes mellitus—T1DM), obesity, autism spectrum disorder, and inflammatory bowel disease [5,6,7,8]. Gut colonization begins at birth, and during the first three years of life, the infant microbiota undergoes dynamic changes. While an adult-like microbial composition begins to emerge around one year of age, a stable community similar to that of adults is typically established by 2.5–3 years [9,10,11]. Research has identified three developmental stages of the early gut microbiota: the developmental stage (3–14 months), dominated by *Bifidobacteria*; the transition stage (15–30 months), characterized by an increase in *Proteobacteria* and *Bacteroidetes*; and the stability stage (≥31 months), where *Firmicutes* become predominant [11,12,13,14].

**Figure 1:**
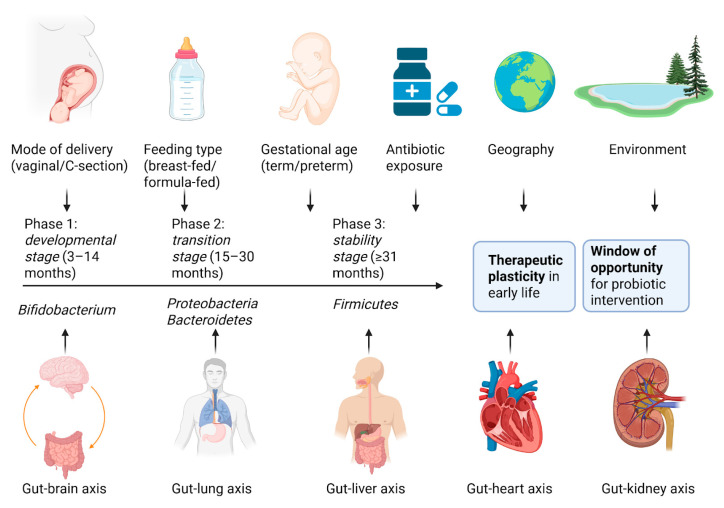
This infographic summarizes the development and influence of the gut microbiota during the first years of life. Key early-life factors—such as delivery mode, feeding type, antibiotic exposure, and environmental conditions—shape microbial colonization from birth. The timeline illustrates a gradual maturation of the microbiota, from low diversity at birth to a stable, adult-like composition by age 3. Gut–organ axes (brain, liver, lung, kidney, heart) represent the systemic impact of microbial signals on long-term health, including risks of allergies, autoimmune diseases, obesity, and neurodevelopmental disorders. Crucially, the infant gut is highly plastic and responsive to interventions, marking early life as a window of opportunity for microbiota-based therapies such as probiotics, prebiotics, or dietary strategies to restore microbial balance and promote lifelong health. Created with BioRender.com (accessed on 4 May 2025).

Several key factors influence the gut microbiota in early life. The mode of delivery is particularly important—vaginally delivered infants show higher levels of *Prevotella* spp. and *Lactobacillus*. In contrast, those born via cesarean section exhibit increased levels of *Corynebacterium*, *Staphylococcus*, and *Propionibacterium* spp. [15]. Feeding type also has a marked impact: breastfed infants tend to harbor more *Lactobacilli* and *Bifidobacteria*, while formula-fed infants display greater abundance of *Bacteroides*, *Clostridia*, *Staphylococci*, *Enterococci*, and *Atopobium* [15,16]. The introduction of solid foods further increases microbial diversity, favoring *Actinobacteria* and *Proteobacteria* while decreasing *Bifidobacteria* [15,17]. Other influential variables include gestational age—preterm infants often present increased levels of *Enterococcus*, *Enterobacteriaceae*, and other opportunistic pathogens—as well as geographical region, family lifestyle (e.g., pet exposure), host genetics, and antibiotic use. For example, Northern European infants typically exhibit higher *Bifidobacterium* levels, whereas Southern European infants have more diverse gut profiles [18]. Genetic polymorphisms, such as those affecting lactase-phlorizin hydrolase expression, also correlate with microbial patterns [15,18,19,20]. Figure 1 synthesizes the development and influencing factors of the gut microbiota during the first years of life.

The gut microbiota communicates bidirectionally with multiple organs via established axes, influencing local and systemic health. The gut–brain axis plays a crucial role in neurodevelopment and microglial function, with microbial metabolites such as short-chain fatty acids (SCFAs) contributing to CNS homeostasis [21]. Pathogens like *Escherichia coli* and *Campylobacter jejuni* have been linked to alterations in behavior and cognitive development [22,23,24]. Emerging evidence also supports an enterothymic communication axis, where early microbial cues influence thymic lymphocyte development via plasmacytoid dendritic cells, with potential long-term implications for immune regulation and colitis risk in adulthood [25,26]. The gut–lung axis modulates respiratory health, with SCFAs reducing pulmonary inflammation and infections like pneumonia, impairing gut epithelial integrity [26,27]. The gut–liver axis is implicated in early-life obesity, where antibiotic-induced shifts in bile acid metabolism negatively affect energy regulation [28,29,30].

Importantly, the infant gut microbiota is highly plastic and adaptable, providing a window of therapeutic opportunity. Antibiotics, dietary modulation, probiotics, prebiotics, and fecal microbiota transplantation (FMT) are being explored to restore or optimize microbial balance. Among these, probiotics have gained particular attention due to their capacity to promote beneficial bacteria, modulate immune responses, and potentially prevent or mitigate disease [28,31].

## 2. Gut Colonization and Microbial Composition During the First 1000 Days of Life

The microbial colonization and immune system development are shaped during the first 1000 days of life, from the fetal period until two years of age—a critical window characterized by high plasticity that plays a pivotal role in long-term health outcomes [32]. In the first days after birth, bidirectional communication between the immune system and the microbiota is essential for maintaining microecological balance and immune homeostasis [32,33]. During pregnancy, the maternal oral, intestinal, and vaginal microbiota change due to the altered immune status, which enables maternal tolerance of fetal cells and prevents immune rejection. These changes also modulate fetal metabolic responses and contribute to the initial colonization of the fetal microbiota [34,35].

Early gut colonization influences subsequent microbial development with long-term effects through competition for binding sites, nutrient utilization, antimicrobial production, and environmental changes [36]. This colonization is shaped by maternal and infant-related factors, which are explored in the following section, resulting in differences in oral and intestinal microbiota development [37,38]. Some of the earliest colonizers are *Streptococcus* spp., which populate the infant’s mouth—the gateway to the gastrointestinal tract—thereby influencing gut microbiota colonization [39,40]. Other early colonizers include facultative anaerobic bacteria, with *Enterobacterales*, *Enterococci*, and *Staphylococci* dominating in the first weeks, followed by an increased abundance of *Clostridium leptum*, *Bifidobacterium*, and *Bacteroides fragilis* [41]. The introduction of solid foods further shifts the gut microbiota toward dominance by *Firmicutes* and *Bacteroidetes* [42].

Fetal colonization of the oral and gut microbiota begins before birth, via exposure to microorganisms from the placenta, amniotic fluid, and meconium, influenced by the maternal microbiota [32,43]. Changes in the maternal gut microbiota may reach the placenta and amniotic fluid through bloodstream dissemination, mediated by dendritic cells that translocate microbes from the intestinal lumen, ultimately affecting the fetus via the umbilical cord [32,44,45]. Older studies analyzing meconium samples using triplicates for fluorescent in situ hybridization (FISH) with 16S rRNA-targeted probes, qPCR/RT qPCR or 16S rRNA gene pyrosequencing, quantitative PCR, and denaturing gradient gel electrophoresis have detected low bacterial loads, mostly *Staphylococcus*, *Enterobacteriaceae*, *Enterococcus*, *Lactobacillus*, and *Bifidobacterium*, suggesting limited colonization at birth [16,32,46,47]. A 2023 recent study by Qiuwen He et al., using 16S rRNA sequencing on samples from 39 pregnant women, found high levels of *Lactobacillus* and *Curvibacter* in amniotic and vaginal fluids, *Bacillus*, *Escherichia*, and *Shigella* in meconium, *Bacteroides* and *Faecalibacterium* in maternal feces, and *Streptococcus* and *Prevotella* in maternal saliva. Notably, the meconium microbiota was most strongly influenced by the amniotic fluid microbiota [48].

Prenatal antibiotic exposure also affects fetal oral and gut colonization, with downstream effects on the infant immune system [32]. A 2018 study by Zou et al. analyzed fecal samples from 28 preterm infants—12 exposed and 12 unexposed to prenatal antibiotics—on days 7 and 14 postnatally, using 16S rRNA sequencing. The results showed decreased *Bacteroidetes* and *Bifidobacterium* and increased *Escherichia-Shigella* in the antibiotic-exposed group [49]. A 2021 meta-analysis by Grech et al., which reviewed 76 studies examining pre-pregnancy and pregnancy exposures affecting the infant microbiome—including maternal antibiotic and probiotic use, diet, pre-pregnancy body mass index (BMI), gestational weight gain (GWG), diabetes, and mood—found that prenatal antibiotic exposure was associated with a reduction in *Actinobacteria* and an increase in *Firmicutes* and *Proteobacteria* in the fetal microbiota [50].

Although fetal colonization begins in utero, the most impactful moment for microbiota establishment is delivery—either vaginal or cesarean—when the commensal microbiota is seeded [32,51]. Vaginally delivered infants acquire healthier oral and gut microbiota, enriched in *Lactobacillus*, *Prevotella*, *Bifidobacterium*, *Bacteroides*, *Lachnospiraceae*, and *Escherichia coli* [51,52,53,54]. In contrast, cesarean-born infants show a higher abundance of *Staphylococcus*, *Corynebacterium*, *Enterococcaceae*, *Enterobacteriaceae*, *Propionibacterium*, and pathogenic species like *Clostridium perfringens* and *Escherichia coli*, with lower *Bifidobacterium* and *Bacteroides* levels [53,55,56,57,58].

Feeding mode further influences early microbiota development, having long-term effects on the oral and intestinal microbiota [32,59]. From birth to around 6 months, breastfeeding, through human milk oligosaccharides, increases the abundance of *Staphylococcus*, *Streptococcus*, *Bifidobacterium* spp. (*bifidum*, *breve*, *longum*), *Propionibacterium*, and *Lactobacillus*, with *Streptococcus* being dominant [60,61]. Breastfeeding also helps shape the immune system and gut health through the “gut–breast axis”—a bidirectional interaction where maternal gut microbiota influence the composition of breast milk, including its microbial content, immune factors, and metabolites, which in turn shape the infant’s gut microbiome and immune development [53,62,63]. In contrast, formula-fed infants show elevated *Actinomyces* and *Prevotella* and reduced *Bifidobacterium*, which is not fully restored by supplementation [64,65].

**Figure 2:**
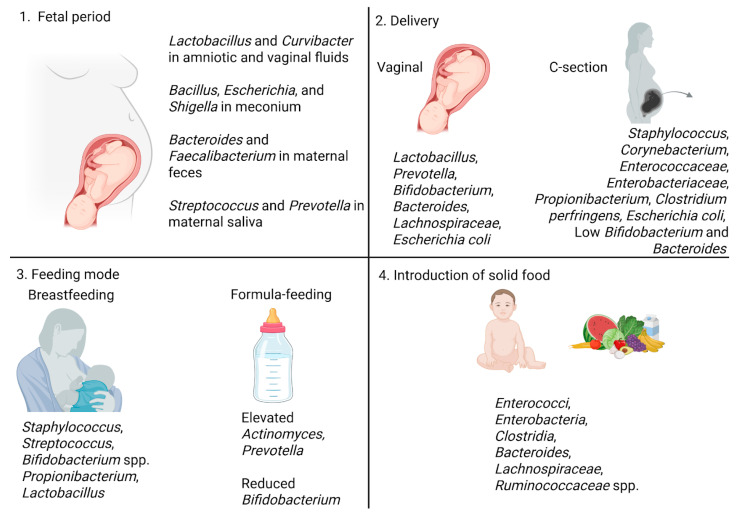
Key stages of microbiota colonization and development in the first 1000 days of life. This infographic highlights the timeline of microbiota establishment from the prenatal period to early childhood, outlining the main influencing factors and the dominant microbial genera associated with each stage. Created with BioRender.com (accessed on 20 May 2025).

The introduction of solid foods increases gut microbiota diversity and promotes communities adapted to fiber and protein, including *Enterococci*, *Enterobacteria*, *Clostridia*, *Bacteroides*, *Lachnospiraceae*, and *Ruminococcaceae* spp. [66,67,68]. A 2020 study by Differding et al. comparing early (<3 months) vs. later solid food introduction and assessing the differential abundance of microbial taxa (measured by 16S rRNA gene sequencing) revealed increased *Bilophila wadsworthia* and *Roseburia* in early-fed infants, associated with elevated butyric acid and linked to immune dysregulation, oxidative stress, and childhood obesity [69]. Another 2017 prospective longitudinal study by Pannaraj et al. that evaluated bacterial composition using sequencing of the 16S ribosomal RNA gene in breast milk, areolar skin, and infant stool samples of 107 healthy mother–infant pairs found that early solids accelerate microbiota maturation, reduce *Bifidobacterium*, and increase adult-associated taxa like *Bacteroides* [70]. Although the microbiota becomes more adult-like by age two, recent evidence suggests that complete maturation continues until adolescence [71,72]. The most important stages in the colonization and development of the microbiota and the main microbial groups during the first 1000 days are illustrated in Figure 2.

Global events such as the COVID-19 pandemic and related control measures may have long-lasting impacts on the human microbiota, including decreased diversity and increased pathogenic bacteria, potentially disrupting maternal–fetal microbiota transmission. This underlines the need for future research focused on supporting healthy oral and gut microbiota development during this critical 1000-day window [32,73].

## 3. Factors Influencing the Early Gut Microbiota

**Figure 3:**
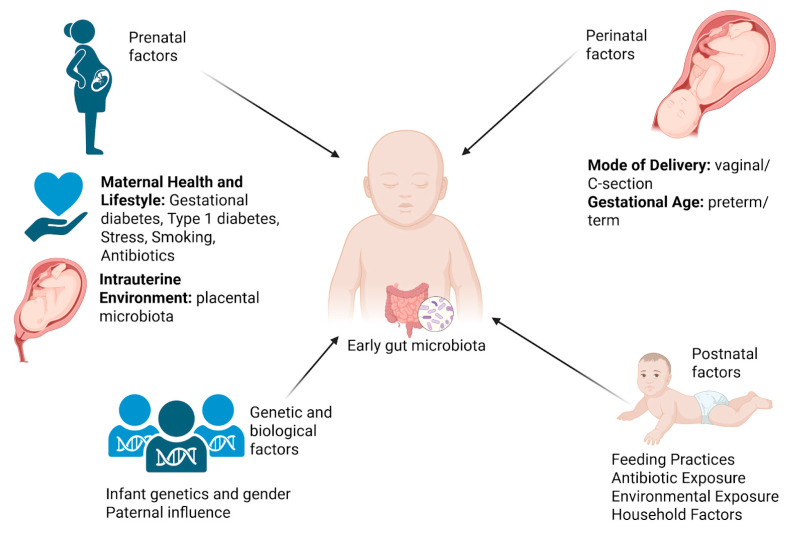
Factors influencing the early gut microbiota. This infographic summarizes the main prenatal, perinatal, and postnatal factors that shape gut microbiota development during the perinatal period. It highlights maternal influences (such as health, diet, stress, and antibiotic use), the mode of delivery, gestational age, feeding practices, early-life antibiotic exposure, environmental and household exposures, and genetic and paternal contributions. Together, these elements influence early microbial colonization, which is critical in shaping immune function, metabolic health, and long-term well-being. Created with BioRender.com (accessed on 23 May 2025).

A convergence of prenatal, perinatal, and postnatal effects contributes to the establishment of the microbiota found in the newborn’s gut. Figure 3 illustrates the key influences on gut microbiota development during the perinatal period.

### 3.1. Prenatal Factors

The early microbial exposure of a newborn can be affected by factors such as the mother’s nutrition, obesity, smoking habits, and antibiotic usage during pregnancy [37]. The mother’s gut microbiota may change as a result of gestational diabetes mellitus (GDM), which may subsequently impact the newborn’s microbiota [74,75]. Women with GDM give birth to babies with different gut microbiota compositions [75,76,77,78]. These changes are characterized by increased concentrations of possibly pro-inflammatory bacteria and less beneficial bacteria like *Lactobacillus* and *Prevotella*. These alterations may put neonates at greater risk for metabolic disorders in the future [79,80,81]. Pregnant women with T1DM exhibit distinct gut microbiota profiles characterized by a decrease in beneficial microbes and an elevation in pro-inflammatory bacteria. These shifts in the perinatal environment may influence the formation of the newborn’s microbiota [82].

A mother’s dietary decisions have a significant influence on the microbiota that is present in her gut, which in turn affects the microbiota that is present in the germs of the developing child. There is a correlation between diets that are high in fiber and low in saturated fat and a more diverse and healthier maternal microbiota, which may result in healthier microbial colonization in infants [83]. This correlation has been shown in studies that have been conducted so far. Compared to other women, the gut microbiota profiles of pregnant women with T1DM are unique. A decrease in beneficial microorganisms and an increase in germs that promote inflammation are the defining characteristics of these cases. There is a possibility that these alterations in the prenatal environment will impact the population of microorganisms generated in the infant [84,85,86].

Variations in the mother’s microbiota have been connected to smoking on the part of the mother. These variations have the potential to influence the microbial colonization of the newborn and raise the likelihood of health problems [87].

Excessive stress levels during pregnancy can influence the microbiota of the mother and the environment inside the uterus, which may have repercussions for the development of the newborn’s microbiota [88,89,90].

The administration of antibiotics by mothers may influence gut microbiota, potentially resulting in a decrease in the diversity of microorganisms. This disruption may affect the early microbial exposure of the baby, potentially impacting the development of the immune system and heightening the risk of certain diseases [55,91].

Although the intrauterine environment was traditionally considered sterile, recent studies have challenged this view, suggesting that microbial DNA may be detected in placental tissues, amniotic fluid, or meconium [47,92,93]. However, the existence of a functional placental microbiota remains controversial, as some findings could be explained by contamination during sampling or sequencing [94,95,96]. Despite this, several studies propose that maternal conditions, such as GDM, may influence the microbial signals in utero and potentially impact early immune and gut development [80,97,98].

### 3.2. Perinatal Factors

Infants that are born vaginally are subjected to the microbiota of their mother’s vaginal and intestinal tracts, which leads to the colonization of beneficial bacteria like Lactobacillus and Bifidobacterium. Neonatal infants who are delivered via cesarean section, on the other hand, have a greater likelihood of being colonized by skin-associated and ambient bacteria, which may hinder the development of their immune systems [99,100].

Premature newborns generally exhibit reduced bacterial diversity and a diminished presence of beneficial bacteria, such as Bifidobacterium, which may influence the development of their immune system and metabolic functions [101]. Gestational age markedly affects the development of the baby’s gut microbiota. Preterm newborns, particularly those born extremely preterm (<28 weeks), demonstrate unique microbial colonization patterns compared to term infants. Compared to term newborns, preterm infants often have a lower variation in microorganisms in their gut. An increasing prevalence of potentially harmful bacteria, such as *Enterobacteriaceae*, and a decreased abundance of beneficial bacteria, such as *Bifidobacterium* and *Bacteroides*, are both positively correlated with the reduced diversity [102,103].

The establishment of gut microbiota in preterm neonates transpires in certain stages, commencing with the predominance of *Staphylococcus*, followed by *Enterococcus*, then *Enterobacter*, and ultimately concluding with *Bifidobacterium*. A link exists between the gestational age of the infant and the duration of each phase, with severely premature infants experiencing prolonged early phases [104,105].

The gut microbiota of preterm infants often aligns with that of term infants by 24 weeks, despite initial differences between the two cohorts. Nonetheless, under certain settings, significant disparities may persist [19].

### 3.3. Postnatal Factors

Breastfeeding promotes the proliferation of a gut microbiota predominantly composed of *Bifidobacterium*. This occurs because prebiotics, specifically human milk oligosaccharides (HMOs), are generated during a mother’s child’s breastfeeding. Infants who receive formula feeding typically exhibit a more diversified microbiota, characterized by a range of distinct bacterial compositions. This is due to the formula introducing a novel array of microorganisms into the environment [28,37].

The administration of antibiotics throughout the early years of life has the potential to disrupt the microbiota that is growing in the gut, resulting in a reduction in the variety of microorganisms and possibly increasing the risk of conditions such as inflammatory bowel disease (IBD) [106,107,108,109,110].

Exposing young children to diverse environmental microbes throughout early infancy can enhance microbial diversity and bolster immune system development. These bacteria may be located in natural habitats or through encounters with pets and siblings. This concept is fundamental to the development of the “biodiversity hypothesis” [111,112,113].

A wider variety of bacteria can be introduced to a child through the presence of older siblings and pets, which has the potential to influence the composition of the microbiota in the infant’s gut tissue [16,114].

### 3.4. Genetic and Biological Factors

The genetic makeup of a newborn can affect the composition of the gut microbiota. There have been studies that have demonstrated that male and female babies exhibit distinct variances in microbial diversity and specific bacterial abundances [115,116].

Recent research indicates that an offspring’s microbiota and health outcomes may be affected by the father’s gut health at the time of conception. This observation suggests that paternal influences contribute to the child’s growth [117].

Emerging research underscores the significant role of paternal gut microbiota in shaping the early-life microbiota and health outcomes of offspring. Mouse studies indicate that disruptions in paternal gut microbiota at conception can negatively impact the offspring. Male mice given antibiotics to alter their gut microbiota exhibited offspring with reduced birth weights, increased postnatal mortality, and placental abnormalities. Alterations in testicular metabolites and hormonal signaling, particularly involving leptin, are implicated, indicating a connection known as a “gut–germline axis” that associates a father’s gastrointestinal health with fertility [118].

Crucially, when the paternal gut microbiota was given time to heal prior to conception, these adverse effects were reversed, indicating that the impacts are temporary and can be lessened by restoring the microbiota.

Additional studies have demonstrated that the father’s gut microbiota might affect his children’s behavior through epigenetic pathways. One study found that sperm short RNAs, essential for gene regulation, changed when male mice’s gut microbiota was reduced. These males’ offspring showed decreased body weight, changed gut shape, and, in the case of females, elevated anxiety and depressive-like behaviors [119].

In addition to genetic and epigenetic influences, fathers directly introduce microorganisms to their offspring. Longitudinal research indicates paternal microbiota can colonize the infant intestine, with the father’s contribution matching the mother’s by the first birthday. In cesarean deliveries, maternal microbial transmission is hindered, rendering paternal seeding essential [120,121].

The relevance of paternal health, particularly the health of the gastrointestinal tract, to reproduction and the well-being of children is brought to light by these latest discoveries. The gut flora of future generations may be influenced by factors such as nutrition, antibiotic medication, and lifestyle decisions that affect the father. Therefore, men interested in conceiving should make sure to keep their gut flora healthy by eating a balanced diet, using antibiotics responsibly, and maintaining general wellness.

## 4. The Gut–Extraintestinal Organ Axes and Later-Life Effects on Human Health

The gut microbiota plays a key role in many physiological processes essential for maintaining homeostasis. These processes include the digestion and absorption of nutrients, the development and regulation of the immune system, and even the modulation of distant organs. Thus, the gut influences host health through complex bidirectional communication networks known as the gut–extraintestinal organ axes. These intricate pathways involve numerous mechanisms and signaling molecules that link the gut to vital organs such as the brain, liver, kidneys, lungs, and heart. Understanding these networks is crucial for a comprehensive appreciation of the gut microbiota’s impact on general health and various diseases [122,123]. The early-life period is essential for properly developing these gut–organ axes. The “Developmental Origins of Health and Disease” hypothesis, which evolved from epidemiological studies of infant and adult mortality, highlights the importance of early environmental exposures in shaping long-term disease susceptibility [124]. The gut microbiota is increasingly recognized as a pivotal mediator within this framework [101].

**Figure 4:**
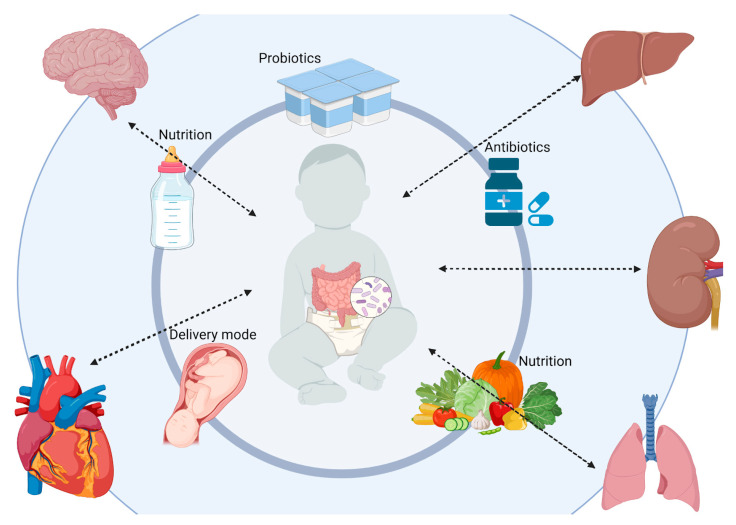
The gut–organ axes. This image illustrates how the early-life gut microbiota establishes critical communication pathways with major organs—the brain, liver, kidneys, and lungs—through what are known as the gut–organ axes. These bidirectional networks are shaped by early environmental exposures like nutrition, antibiotics, and delivery mode, influencing lifelong outcomes such as neurological development, metabolic health, kidney function, and respiratory resilience. Disruptions during this sensitive window can predispose individuals to chronic diseases later in life, highlighting the gut’s foundational role in systemic health. Created with BioRender.com (accessed on 25 May 2025).

The first years of life play a major role in the development of the gut microbiota and the gut–organ networks through dietary and environmental factors [125,126]. Disruptions during this critical period, such as antibiotic administration or suboptimal nutrition, can lead to dysbiosis, which is defined as imbalances in the gut microbial community. Figure 4 illustrates the bidirectional networks between the gut and extraintestinal organs and their early environmental factors.

### 4.1. The Gut–Brain Axis and Later-Life Neurological Health

The gut–brain axis, which integrates the GI tract and the central nervous system (CNS), is a prime example of these intricate bidirectional communication systems. This network facilitates a continuous exchange of information between the brain and the gut, influencing several physiological and behavioral processes crucial for maintaining homeostasis [122]. The gut microbiota modulates this axis, impacting brain development, behavior, and neural functions [123].

Several interconnected pathways, such as neural, endocrine, immune, or metabolic pathways, facilitate communication within the gut–brain axis. Neural pathways, mainly via the vagus nerve, serve as bidirectional routes for transmitting signals between the gut and the brain, modulating brain activity and bodily functions [123]. The enteric nervous system (ENS) is a complex network of neurons found in the gut wall that communicates with the CNS, modulating motility, secretion, and other functions [127]. Endocrine pathways involve the secretion of numerous gut hormones, such as glucagon-like peptide (GLP1) and peptide YY, which enter the bloodstream and exert effects on brain regions involved in functions such as appetite, mood, and cognition [123].

Another key communication pathway between the brain and the gut is represented by the hypothalamic–pituitary–adrenal axis, a central component of the body’s stress response system [128]. Immune pathways are also extensively involved, and the gut microbiota plays a key role in developing and regulating the host’s immune system. Dysbiosis can trigger the production of pro-inflammatory cytokines and other immune mediators that can cross the blood–brain barrier or influence its permeability [129]. This can lead to neuroinflammation, which plays a major role in several neurological disorders [130].

Metabolic pathways are also critical, with microbial metabolites, particularly SCFAs produced through the fermentation of dietary fibers by gut bacteria, acting as key signaling molecules along the gut–brain axis, influencing the immune system, epigenetics, and neuroplasticity in the CNS [131]. Imbalances in the tryptophan metabolism via the serotonergic and kynurenine pathways have been linked to several CNS pathologies, such as dementia, Huntigton’s disease, and Alzheimer’s disease (AD) [132]. Dysregulations in this pathway can lead to imbalances between neurotoxic and neuroprotective factors, potentially contributing to neurodegeneration [132,133]. Furthermore, the gut microbiota has the remarkable ability to produce a variety of neuroactive substances, including neurotransmitters such as serotonin, dopamine, and gamma-aminobutyric acid (GABA), which can directly or indirectly influence brain function and behavior [130]. Notably, this communication is inherently bidirectional, with the brain also exerting significant control over gut physiology, including motility, secretion, and immune responses, which can profoundly influence the composition and functional activity of the gut microbiota.

Emerging research has established a relationship between alterations in the early-life gut microbiota and the development of several neurodevelopmental disorders, including Autism spectrum disorder (ASD) [101]. Numerous studies have observed differences in the gut microbiota populations of children with ASD compared to neurotypical children, including differences in the abundance of bacterial genera such as *Bacteroides*, *Clostridium*, *Lactobacillus*, and *Bifidobacterium* [101]. In addition, preclinical studies have suggested that targeted interventions aimed at modulating the gut microbiota, such as administering *Limosilactobacillus reuteri* probiotics, may improve social behavior in children diagnosed with ASD. Moreover, several studies have demonstrated that the administration of multispecies probiotics during pregnancy can have beneficial effects on the neurodevelopment of children [101].

Similarly, the early-life gut microbiota has been increasingly implicated in the etiology and progression of Attention-Deficit/Hyperactivity Disorder (ADHD) [128]. Research has demonstrated that alterations in the infant gut microbiota composition, including a reduction in phylogenetic diversity and specific changes in the abundance of bacterial taxa belonging to the order *Lactobacillalus* and the genus *Bifidobacterium*, are associated with the subsequent development of ADHD [134]. In addition, other studies have shown that the microbiota of ADHD patients is characterized by decreased diversity and bacterial richness [135,136]. The genera dominating the gut microbiota population of subjects with ADHD are represented by *Agathobacter*, *Akkermansia*, *Anaerostipes*, *Bifidobacterium*, *Blautia*, *Collinsella*, *Dorea*, *Eggerthella*, *Escherichia/Shigella*, *Fusobacterium*, *Intestinibacter*, *Megamonas*, *Neisseria*, *Odoribacter*, *Phascolarctobacterium*, and *Roseburia* according to one review paper [135]. Moreover, a randomized controlled trial involving the supplementation of *Lactobacillus rhamnosus* GG during the first six months of life reported a complete absence of ADHD diagnoses in the probiotic-treated group by the age of 13 years, suggesting a potential protective role for early probiotic intervention [137].

In addition, alterations in the early-life gut microbiota have also been observed in association with other neurodevelopmental disorders, including Tourette Syndrome, Cerebral Palsy, and Rett Syndrome [135]. For instance, patients suffering from Rett Syndrome often exhibit altered gastrointestinal homeostasis and specific changes in the abundance of bacterial genera such as *Actinomyces*, *Bifidobacterium*, and *Clostridium* [138].

Recent research also suggests a potential link between early gut health and neurodegenerative diseases in adults, such as AD and Parkinson’s disease (PD) [139,140]. Although longitudinal studies are lacking, cross-sectional studies and animal studies have shown that there is a relationship between dysbiosis and neurodegenerative diseases [101]. Disruptions in the gut microbiota in early life lead to low-grade inflammation and altered production of neuroactive metabolites, which may contribute to an increased vulnerability of the brain to age-related neurodegenerative processes [101]. The gut–brain axis is believed to be involved in the pathogenesis of both AD and PD, with alterations in the gut microbiota potentially influencing neuroinflammation, the accumulation of amyloidogenic proteins in AD, and the misfolding of α-synuclein in PD, via increased trimethylamine N-oxide (TMAO) levels [141]. The concept of “inflammaging,” a state of chronic, low-grade inflammation associated with aging, may provide a mechanistic link between early gut microbial imbalances and the later development of neurodegenerative conditions [142].

Recent research has begun to elucidate how the gut microbiota and metabolome influence early neurodevelopmental processes. Animal studies have demonstrated that the hippocampal development is greater in germ-free mice. The transfer of gut microbes from conventional mice did not affect the process, suggesting that the gut microbiota influences neurogenesis from an early stage. In addition, researchers have demonstrated that the administration of specific microbial strains can counteract deficient neurogenesis [143].

### 4.2. The Gut–Liver Axis and Long-Term Metabolic Health

The gut–liver axis represents an important bidirectional communication pathway that involves the gut microbiota, the intestinal barrier, the portal vein, and the liver. The early-life microbiota highly influences the liver’s development and function. Gut microbes and their diverse array of metabolites are transported directly to the liver via the portal vein, which influences hepatic lipid metabolism, glucose homeostasis, and immune responses within the liver [144].

Structural and functional differences in the neonatal gut barrier influence gut–liver interactions and may shape long-term immune and metabolic health. For example, in full-term infants, the small intestinal epithelium is already organized into crypts and villi, unlike in mammals such as mice, where this structure forms after birth. Neonates also show increased macromolecular uptake, facilitated by mechanisms like FcRn-mediated IgG transport and immature tight junctions, which support early immune development but decline after weaning [145].

The gut–liver axis is influenced by early-life environmental exposures, including nutrition, pollutants, and microbiota composition, which can epigenetically reprogram this bidirectional pathway, with lasting effects on metabolic health. Since the gut, liver, and immune system are still maturing during early life, this period is especially sensitive to environmental influences that can shape long-term health. One example is neonatal exposure to polybrominated diphenyl ether-99 (BDE-99), a persistent flame retardant commonly detected in breast milk. Studies on rodents have shown that early-life exposure to BDE-99 alters hepatic gene expression patterns, particularly those involved in lipid metabolism and inflammatory signaling, ultimately promoting a pro-inflammatory metabolic phenotype in male adults, via increased intestinal levels of *Akkermansia muciniphila* and its metabolites acetate and succinate [146]. This demonstrates that harmful environmental chemicals can modulate the liver metabolism, possibly by affecting gut bacteria, how the gut lining works, or how signals travel between the gut and liver.

The gut microbiota composition is highly influenced by maternal nutrition during pregnancy and by the delivery mode [147]. Moreover, early microbial communities regulate host energy storage and bile acid metabolism, both main components of the gut–liver axis. For instance, a 2015 study by Bäckhed et al. that evaluated the fecal samples from a cohort of 98 Swedish infants and their mothers using metagenomic analysis demonstrated that germ-free mice colonized with gut microbiota showed increased fat storage and altered bile acid profiles, highlighting how microbial signals can direct metabolic outcomes [51]. Recent studies have also linked gut dysbiosis associated with maternal obesity during early life to increased intestinal permeability and the initiation of pathological pathways that can lead to the development of nonalcoholic fatty liver disease [148]. Importantly, the liver also contributes to gut homeostasis, completing the bidirectional nature of this axis. Bile acids synthesized in the liver are secreted into the intestine, where they aid in lipid absorption and shape the composition and function of the gut microbiota by acting as signaling molecules through receptors such as FXR and TGR5. Additionally, hepatically derived immune mediators and metabolic products can influence intestinal epithelial integrity and immune responses, thereby maintaining gut barrier function and microbial balance [149]. Altogether, these early-life influences on the gut–liver connection can shape how the immune system develops and increases the risk of health problems later on, including obesity, fatty liver disease, and type 2 diabetes. Establishing a healthy gut microbiota in early life is likely crucial for proper liver development and long-term metabolic health [148].

### 4.3. The Gut–Kidney Axis and Long-Term Renal Health

The gut–kidney axis represents a bidirectional communication pathway between the gastrointestinal tract and the kidneys, primarily mediated by the gut microbiota and metabolites. This intricate interplay is crucial for maintaining homeostasis and has gained attention for its role in the pathogenesis of kidney diseases, including chronic kidney disease (CKD) and acute kidney injury [150]. As observed in the other bidirectional axes between the gut microbiota and vital organs, early-life factors such as mother nutrition during pregnancy, delivery mode, breastfeeding, antibiotic exposure, and environmental factors highly influence gut microbiota development and thus the gut–kidney axis [151]. Disruptions during early life can lead to dysbiosis and predispose individuals to kidney dysfunction later in life [152]. Some of the main modulators of gut health are SCFAs, metabolites produced via the metabolization of fiber products, which play a major role in maintaining gut barrier integrity and function and play a protective role in modulating immune responses [152]. Dysbiosis can compromise intestinal barrier integrity and lead to increased gut permeability, also known as “leaky gut”. This condition allows the translocation of endotoxins such as lipopolysaccharides into the systemic circulation, triggering systemic inflammation, which can lead to kidney injury via pro-inflammatory and oxidative pathways [150]. In addition, an altered gut microbiota can produce uremic toxins like indoxyl sulfate and p-cresyl sulfate, which accumulate in CKD due to reduced renal clearance, exacerbating oxidative stress and inflammation [153]. Another interaction mechanism between the gut and the kidney is represented by advanced glycation products (AGEs). AGEs, formed endogenously or through high-heat cooking and processing of foods, accumulate in CKD due to impaired clearance. By binding to RAGE (receptor for advanced glycation end products), AGEs trigger inflammation, oxidative stress, and fibrosis, worsening renal damage and contributing to gut permeability and vascular dysfunction. Reducing dietary AGE intake may offer a therapeutic approach to slowing CKD progression by mitigating systemic inflammation and kidney injury [150].

One recent article showed how the microbiota, via the modulation of systemic inflammation and metabolic regulation, interacts with adipose tissue, particularly brown adipose tissue, to impact kidney function. Disruptions in the gut microbiota can impair brown adipose tissue activity, reduce energy expenditure, and exacerbate glucose dysregulation, all of which contribute to diabetic kidney disease progression [154].

Conversely, kidney dysfunction can negatively impact gut homeostasis, reinforcing the bidirectional nature of this axis. In chronic kidney disease, uremia alters the intestinal environment, leading to changes in pH, an increased urea concentration in the gut lumen, and compromised epithelial barrier function. These changes promote dysbiosis, reduce beneficial SCFA-producing bacteria, and enhance the growth of pathogenic species. Additionally, systemic inflammation and accumulation of uremic toxins in CKD can disrupt intestinal tight junctions and increase gut permeability, further amplifying the cycle of gut–kidney dysfunction [155,156].

Therapeutic strategies targeting the gut–kidney axis are being explored, including prebiotics, probiotics, and dietary modifications for restoring a healthy microbiota balance. Early-life interventions, such as promoting breastfeeding and cautious use of antibiotics, may also play a role in establishing a beneficial gut microbiota, potentially reducing the risk of kidney diseases in later life [150].

In conclusion, the gut–kidney axis is a pivotal component in the development and progression of kidney diseases. Understanding its mechanisms, especially during early life, opens avenues for preventive and therapeutic strategies to preserve renal health through the modulation of the gut microbiota.

### 4.4. The Gut–Lung Axis and Long-Term Respiratory Health

The gut–lung axis describes the bidirectional communication pathway between the gut and the lungs, involving the gut microbiota and the pulmonary system. The lung and gut microbiotas are integral to maintaining respiratory and gastrointestinal health, respectively, and they engage in dynamic interactions through the gut–lung axis. The lung microbiota was initially thought to be sterile. However, recent research has shown that it comprises diverse microbial communities that influence pulmonary immunity and disease susceptibility [157]. Similarly, the gut microbiota plays a crucial role in immune system development and function [158]. These two microbiotas communicate bidirectionally via immune signaling pathways and microbial metabolites, such as SCFAs, which can modulate inflammatory responses in the lungs and the gut. Disruptions in either microbiota can lead to dysbiosis, contributing to the development and progression of diseases such as asthma, chronic obstructive pulmonary disease, and inflammatory bowel disease [159]. Understanding the interplay between the lung and gut microbiotas is essential for developing holistic approaches to preventing and treating these interconnected conditions.

The early-life gut microbiota plays a pivotal role in the development and maturation of the host’s immune system, which has profound implications for respiratory health throughout the lifespan [159]. The initial colonization of the gut, established within the first one to three years of life, plays a critical role in shaping the lung immune system and fostering immune tolerance to airborne antigens. This early period is considered a “critical window” for immune programming that can affect global health status for a lifetime, including susceptibility to respiratory diseases [160].

Dysbiosis associated with disruptions in the gut microbiota during this crucial early period has been consistently associated with an increased risk of developing various respiratory conditions [161]. For example, a systematic review of human studies found evidence that low alpha-diversity and reduced relative abundance of specific gut–commensal bacteria genera, including *Bifidobacterium*, *Faecalibacterium*, *Ruminococcus*, and *Roseburia*, are associated with childhood respiratory diseases such as respiratory infections, recurrent wheezing, and asthma [162]. Specifically, research has shown a correlation between low levels of intestinal *Bifidobacterium* microbiota during infancy and an increased susceptibility to atopy, a predisposition to allergic reactions, including asthma, later in life [163].

The mode of delivery is known to play a crucial role in the development of the gut microbiota, and it has been shown that infants born by cesarean section tend to have a higher abundance of opportunistic pathogens during the neonatal period compared to those born vaginally [54]. This disturbance in early microbiota development has been suggested to contribute to an increased risk of immune-mediated conditions like asthma and allergies later in life [164]. Beyond asthma, early-life gut dysbiosis has also been linked to other severe respiratory conditions, such as bronchopulmonary dysplasia (BPD), a chronic lung disease affecting premature babies [165]. Studies have observed lower diversity of airway microbiota and specific shifts in bacterial populations (e.g., decreased *Firmicutes* and *Fusobacteria*, increased *Proteobacteria*) in infants with BPD [166].

Emerging evidence also suggests lung inflammation and respiratory infections can reciprocally influence gut homeostasis. Inflammatory cytokines released during lung infections, such as influenza or pneumonia, can enter the systemic circulation and alter gut permeability, epithelial function, and microbiota composition. For example, animal studies have shown that lung inflammation reduces the expression of tight junction proteins in the gut and decreases SCFA-producing bacteria, promoting gut dysbiosis. In addition, respiratory viral infections such as SARS-CoV2 have been associated with gastrointestinal symptoms and gut microbiota alterations, highlighting how pulmonary immune responses can extend their impact to the intestinal environment [167,168,169].

Given the profound impact of early-life gut microbiota on respiratory health, interventions aimed at modulating the gut microbiota, such as the administration of probiotics, are being explored as potential strategies to prevent or mitigate these conditions. Probiotics, particularly species from the *Lactobacillus* and *Bifidobacterium* genera, are recognized for their role in restoring microbial balance and have shown promise in maintaining the balance of microorganisms in the respiratory tract, suggesting a potential therapeutic avenue through the gut–lung axis [166,170].

### 4.5. The Gut–Heart Axis and Long-Term Cardiovascular Health

Mounting research suggests that our cardiovascular health may begin in the gut. The heart and gastrointestinal tract are connected through a dynamic network of microbial, immune, and metabolic signals, known as the gut–heart axis. This intricate network begins developing early in life, as the gut microbiota takes shape during the first months after birth. As previously observed, the microbial communities that establish during infancy not only are influenced by factors like birth mode, early feeding practices, and antibiotic exposure, but also leave lasting marks on host metabolism and immune regulation. When imbalances occur during critical developmental periods, it may set the stage for inflammation, metabolic dysfunction, and increased cardiovascular risk later in life [171,172].

Recent advances in metagenomics and metabolomics analysis have enabled researchers to identify specific gut microbiota profiles and microbial-derived metabolites that contribute to the development of various cardiovascular diseases [172,173]. In particular, shifts in the gut microbiota composition, such as an altered Firmicutes to Bacteroidetes (F/B) ratio, have been consistently associated with metabolic disturbances contributing to cardiovascular disease. Increased levels of *Firmicutes* have been linked to increased nutrient uptake and low-grade inflammation, while a reduction in *Bacteroidetes* can disrupt the production of beneficial microbial metabolites [174]. This imbalance affects key compounds like SCFAs, which normally help regulate blood pressure, vascular tone, and immune responses. At the same time, the microbial conversion of dietary nutrients like choline and L-carnitine into TMAO has emerged as a critical pro-atherogenic pathway, with higher TMAO levels being predictive of adverse cardiovascular outcomes in both animal models and humans [175,176]. These metabolic shifts, driven by early-life microbial development, underscore the gut microbiota’s role as a key determinant in long-term cardiovascular health. Elevated TMAO levels are linked to increased platelet reactivity, vascular inflammation, and atherosclerotic plaque development [177,178]. Bacteria such as *Clostridium* spp. and *Escherichia* spp. are known TMA producers and have been associated with detrimental cardiovascular outcomes [179]. Conversely, certain microbial taxa have protective cardiovascular effects. For example, bacteria that produce SCFAs, such as *Faecalibacterium prausnitzii*, *Roseburia* spp., and *Akkermansia muciniphila,* have been associated with anti-inflammatory effects, improved endothelial function, and lower blood pressure [180,181,182]. SCFAs maintain gut barrier integrity and modulate systemic immunity and metabolic signaling, offering protection against cardiovascular diseases, such as hypertension and atherosclerosis [178,183]. Research has shown that increased dietary fiber intake, especially early in life, fosters these SCFA-producing bacteria and is linked to lower long-term cardiovascular risk [182].

Animal studies have helped clarify causal relationships. For example, germ-free mice colonized with microbiota from hypertensive human donors develop higher blood pressure themselves, directly linking microbial composition to disease traits [184]. Similarly, microbiota from individuals with atherosclerosis have been shown to increase plaque development in animal models [185]. These findings are now being mirrored in human cohort studies, such as the Framingham Heart Study, which identified microbial pathways involving the *Oscillobacter* genus, which plays a major role in cholesterol metabolism and is associated with heart disease [186].

Early life significantly impacts the development of these microbial patterns, which early interventions can shape. Breastfeeding, for example, promotes the growth of *Bifidobacteria*, which have anti-inflammatory properties and support mucosal immunity. On the other hand, early-life antibiotic exposure or formula feeding may favor the development of pro-inflammatory microbes, setting the stage for metabolic and cardiovascular dysfunction later in life [179]. Thus, the gut–heart axis represents a promising frontier for understanding and potentially preventing cardiovascular disease. A balanced, fiber-rich diet starting early in life and strategies to support a healthy microbiota may help reduce lifetime cardiovascular risk. Continued research is needed to understand fully how gut microbes and their metabolites interact with the cardiovascular system over a lifespan.

## 5. Modulating Gut Microbiota During Perinatal Life

The child’s microbiota develops and matures throughout the perinatal period, significantly influencing the newborn’s future health. Variables including the mode of delivery, maternal nutrition, antibiotic exposure, and infant feeding practices significantly influence the composition and diversity of the newborn gut microbiota. The first microbial communities significantly influence infants’ immune systems, metabolism, and neurodevelopment.

During pregnancy, the mother’s microbiota alters in a variety of ways. These changes are being made to accommodate and sustain the developing baby while preparing the body for birthing. The first findings of the research suggest that a microbiota adaptation transpires during pregnancy, marked by an augmentation in bacterial diversity [187,188].

Because each bacterium in the microbiota has a distinct role in maintaining health and equilibrium, the variety of the microbiota is critical to its functioning. The gut microbiota is dynamic; it evolves during a person’s lifespan, starting at birth and continuing throughout their whole life [189,190].

The microbiota that lives in the gut is very important to human health because it has a substantial impact on the maturation of the immune system, metabolic programming, the formation of the intestinal barrier, brain development, and long-term health effects [122,123,191,192,193].

The enormous and complex ecosystem of the human abdominal cavity may include a basic collection of microbial species known as the native core microbiota. This collection of microbes is expected to be found inside the human abdominal cavity [194]. This essential assemblage of bacteria begins to inhabit the gut at a young age, establishing the foundation for an individual’s microbiota composition [194].

Recent studies challenge the notion of a universal “core microbiota” shared equally among all humans. While certain bacterial taxa—such as Bacteroidetes and Firmicutes—are commonly found across populations, their relative abundances vary widely, and many species are unique to individuals depending on their diet, lifestyle, and genetics [195,196,197,198]. Longitudinal analyses show that even within a single person, gut microbial composition fluctuates over time (intra-individual variation, ~20–40%), though this is substantially smaller than the differences observed between different individuals (inter-individual variation, ~75%) [199]. Genetic factors, including human gene variants such as ABO or FUT2, further shape microbiota composition in specific taxa like *Faecalibacterium prausnitzii*. Therefore, rather than a one-size-fits-all core microbiota, each person harbors a dynamic and personalized microbial signature, influenced by environment, diet, and host biology [198,200].

Immunological and metabolic programming occur during the perinatal period, when the native core microbiota plays a pivotal role. Ten thousand nine hundred thirty-five shotgun metagenomic datasets were analyzed using taxonomical and functional classifications to investigate microbial diversity over the first three years of human life. The distribution of microbiological species in infants is regulated by variables like their health status and area of origin. Among the eight main Infant Community State Types, 17 bacterial species were predominant [201].

A variety of variables impact the formation of the core microbiota in neonates, most notably the mother’s microbiota, the delivery process, and the nourishment provided to the newborn [201,202].

Full-term newborns generally exhibit greater gut microbiota diversity than preterm infants, which underscores the strong influence of birth conditions such as gestational age, the mode of delivery, and early-life exposures. For instance, in a 2019 study by Fouhy et al. that sequenced DNA from fecal samples of 159 children over the first four years, the authors demonstrated that gestational age significantly impacts microbiota composition up to four years of age, with term-born infants showing more stable, diverse microbial communities compared to their preterm counterparts [202]. While interventions like breastfeeding and minimizing antibiotic use confer clear early benefits—including promoting beneficial taxa such as *Bifidobacterium* and *Lactobacillus*—emerging evidence suggests that well-timed dietary and probiotic interventions may have an even greater long-term impact. For example, corrections applied in infancy via probiotic supplementation during antibiotic exposure or following caesarean birth helped reshape microbial composition toward patterns seen in healthy term infants [203,204,205,206,207]. Moreover, the core microbiota influences metabolic programming, potentially modifying an individual’s susceptibility to metabolic disorders such as obesity and diabetes in later life. The microbiota influences energy balance and metabolic processes throughout time [208].

When it comes to the establishment of the native core microbiota, the perinatal period is marked by greater vulnerability, and any disruptions that occur during this phase have the potential to yield long-term health implications [209,210,211].

The mode of administration has a considerable impact on the microbiota of the newborn gut. Techniques such as maternal vaginal seeding have lately been investigated as potential means of mitigating the impact of this effect. The administration of mother vaginal fluids to neonates who were delivered by the cesarean section is known as maternal vaginal seeding. This technique can resolve the disparities in the development of microbiota that exist between infants who were born vaginally and those who were born after cesarean delivery was performed [212,213,214,215].

Taking extra probiotics while pregnant has been shown to help the mother’s vaginal bacteria. It was clear that this action greatly reduced the number of dangerous germs, creating a better microbiota for the baby. Still, there is not much proof that this action changes the variety of microbiotas in newborns or sets up long-term infection [216,217,218,219,220].

The microbiota of formula-fed infants may resemble that of older infants in composition; however, it may lack the beneficial bacteria present in breast milk [221,222].

The absence of HMOs and other bioactive chemicals in formula may lead to a unique pattern of microbial colonization and metabolic activity in the gastrointestinal system [223]. HMOs are one of the most important prebiotics for keeping an infant’s gut bacteria healthy. By adding these oligosaccharides to baby formula, the benefits of breast milk have been copied [224,225].

The baby’s immune system and metabolic setting could be affected by changes in microbial contact and the lack of biological protection from breast milk. To make up for the difference between breast milk and formula, enriched formulas with HMOs, probiotics, prebiotics, and postbiotics have been made. The goal is to replicate the gut bacteria of nursing children and support strong immune development [223,226,227,228].

The delivery process significantly impacts the baby’s microbiota’s first colonization. There are significant variations between vaginal births and cesarean deliveries (C-sections). During vaginal birth, the baby may be exposed to microbiota from the mother’s vagina and feces. This encourages introducing beneficial bacteria, such as *Lactobacillus* and *Bifidobacterium* species, into the infant’s digestive tract [212,229,230].

The growing body of research sheds insight into the potential for targeted therapeutics to improve microbiota development. These therapies might include dietary modifications, probiotic and prebiotic supplements, and innovative approaches.

## 6. Future Considerations Regarding Intestinal-Microbiota-Based Therapeutic Methods in Pediatric Disease

**Figure 5:**
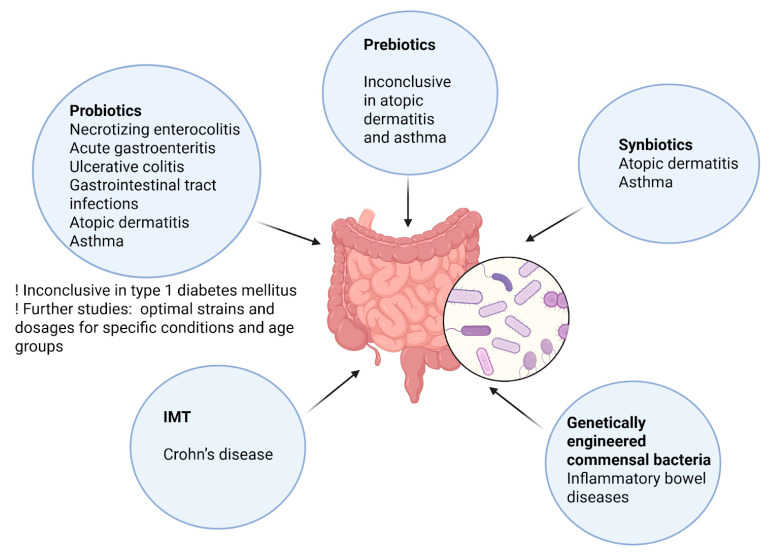
Microbiota-based therapies in pediatric health. This infographic summarizes emerging microbiota-based therapies used to prevent and manage pediatric diseases linked to gut dysbiosis. It highlights the roles of probiotics, synbiotics, prebiotics, IMT, and genetically engineered bacteria in treating conditions such as atopic dermatitis, asthma, gastrointestinal infections, Crohn’s disease, type 1 diabetes, etc. It also emphasizes the importance of selecting specific microbial strains and optimizing treatment protocols and the potential of innovative strategies like microbial drug delivery. Created with BioRender.com (accessed on 25 May 2025).

The burgeoning field of microbiota-based therapies offers promising avenues for addressing pediatric diseases associated with gut dysbiosis. Recent research highlights the potential of probiotics, synbiotics, postbiotics, and intestinal microbiota transplantation (IMT)—formerly known as FMT—in modulating the gut microbiota to improve health outcomes in children [231,232]. Figure 5 summarizes emerging microbiota-based therapies used to prevent and manage pediatric diseases linked to gut dysbiosis.

Probiotics have demonstrated efficacy in managing various gastrointestinal conditions in children. A narrative review including 39 studies (25 single-strain and 14 multi-strain) found that both formulations effectively treated disorders such as NEC, acute gastroenteritis, and ulcerative colitis in pediatric patients under 18. Multi-strain probiotics showed consistently positive outcomes, with a low incidence of adverse effects for both types. These findings highlight the need for further studies to determine the optimal strains and dosages for specific conditions and age groups, ultimately integrating probiotics into clinical practice to reduce the incidence and severity of gastrointestinal disorders [233]. In gastrointestinal tract infections (GITIs), a systematic review and meta-analysis reported that probiotic supplementation reduced the risk of GITI episodes by 26% among children attending childcare centers. Specific strains, such as *Lacticaseibacillus paracasei*, *Limosilactobacillus reuteri*, and *Lacticaseibacillus rhamnosus* GG, were particularly effective, while the effects of *Bifidobacterium animalis* subsp. *Lactis* BB-12 require further study [233,234].

Meta-analyses assessing the impact of prebiotics, probiotics, and synbiotics on pediatric atopic dermatitis have shown mixed results. Some studies demonstrated beneficial effects, while others did not find significant differences [235,236,237,238]. However, a more recent umbrella meta-analysis that included 38 meta-analyses concluded that specific strains of pre-, pro-, and synbiotics could significantly reduce the incidence and severity of atopic dermatitis (RR = 0.74, 95% CI: 0.70–0.79). Multi-strain probiotics, *Lactobacillus* species, and synbiotic formulations showed the greatest efficacy in reducing SCORAD (Scoring Atopic Dermatitis) indices, whereas *Bifidobacterium* and prebiotics alone did not yield significant improvements [239].

The gut–lung axis is increasingly recognized in the pathogenesis of asthma. An expanding body of research is mapping the normal composition and influencing factors of lung and gut microbiota across developmental stages better to understand their roles in asthma phenotypes and endotypes [240]. A systematic review of 18 randomized controlled trials found that probiotics—particularly strains of *Lactobacillus* and *Bifidobacterium*—reduced asthma exacerbations and improved pulmonary function in children. Synbiotics decreased the incidence of viral respiratory infections and healthcare utilization, while postbiotics (including bacterial lysates) were shown to attenuate airway hyperresponsiveness and systemic inflammation [231,241,242,243,244]. The proposed mechanisms include immune modulation and the suppression of Th2 cytokines [245,246]. Despite these findings, the effects of prebiotics on pediatric asthma remain under-investigated and warrant further study [231].

FMT has emerged as a potential therapy for pediatric Crohn’s disease. In a prospective trial involving 33 children with active Crohn’s disease, those treated with IMT capsules combined with partial enteral nutrition showed improved clinical outcomes and restored gut microbiota diversity compared to those receiving partial enteral nutrition with immunosuppressants. Post-treatment analysis revealed increased levels of beneficial genera such as *Agathobacter*, *Akkermansia*, *Roseburia*, *Blautia*, *Subdoligranulum*, and *Faecalibacterium*, along with elevated interleukin-10 (IL-10), an anti-inflammatory marker. These microbial changes may serve as future biomarkers for disease identification and monitoring [247].

An innovative therapeutic approach involves using genetically engineered commensal bacteria to deliver therapeutic agents directly to the gastrointestinal tract, a method known as microbial drug delivery. This strategy has shown promise in treating IBD by enabling localized production of anti-inflammatory molecules, thereby reducing systemic side effects [248]. For instance, *Lactococcus lactis* has been engineered to secrete interleukin-10, an anti-inflammatory cytokine. In murine models, oral administration of interleukin-10-producing *L. lactis* led to a significant reduction in colitis symptoms, demonstrating the potential of this approach for localized therapy [249].

In the case of T1DM, the interplay between gut microbiota and metabolic regulation is becoming increasingly evident. While standard treatment involves insulin therapy, the role of microbiota-based interventions is being explored. A systematic review and meta-analysis involving 388 pediatric and adolescent patients found no statistically significant improvement in glycemic control following probiotic supplementation. A trend toward worsened glycemic outcomes was observed, underscoring the need for better-designed studies with extended follow-up and appropriate strain selection [250]. However, a randomized, placebo-controlled pilot study by Kumar et al. involving 90 newly diagnosed T1DM children showed that a multi-strain probiotic significantly decreased HbA1c levels and insulin requirements over a 3-month period [251]. In contrast, another randomized controlled trial by Groele et al. found that supplementation with *Lactobacillus rhamnosus* GG and *Bifidobacterium lactis* Bb12 did not preserve residual beta-cell function in children newly diagnosed with T1DM [252].

Integrating microbiota-based therapies into pediatric healthcare holds significant promise for preventing and managing a wide range of dysbiosis-related diseases. Ongoing research is essential to determine optimal microbial strains, dosages, and treatment durations, as well as to elucidate the long-term implications of such interventions. Collaborative efforts between researchers, clinicians, and regulatory agencies will be crucial in translating these findings into safe, effective, and widely accessible clinical practices.

## Abbreviations

The following abbreviations are used in this manuscript:

AD	Alzheimer’s disease
ADHD	Attention-Deficit/Hyperactivity Disorder
AGEs	advanced glycation products
ASD	autism spectrum disorder
BMI	body mass index
BPD	bronchopulmonary dysplasia
CKD	chronic kidney disease
CNS	central nervous system
ENS	enteric nervous system
FISH	fluorescent in situ hybridization
FMT	fecal microbiota transplantation
GABA	gamma-aminobutyric acid
GDM	gestational diabetes mellitus
GLP1	glucagon-like peptide
GWG	gestational weight gain
HMOs	human milk oligosaccharides
IBD	inflammatory bowel disease
IMT	intestinal microbiota transplantation
NEC	necrotizing enterocolitis
PD	Parkinson’s disease
RAGE	receptor for advanced glycation end products
SCFAs	short-chain fatty acids
T1DM	type 1 diabetes mellitus
TMAO	trimethylamine N-oxide
